# Supplementary material for: Extraction of Naringin from Pomelo and Its Therapeutic Potentials against Hyperlipidemia
Source: Molecules. 2022 Dec 18;27(24):9033. doi: 10.3390/molecules27249033 (PMC9783781; doi:10.3390/molecules27249033)
Supplement: Supplementary file 1 [file molecules-27-09033-s001.zip › molecules-2050393-supplementary.pdf]

Supporting Information

Extraction of naringin from Pomelo and its therapeutic potentials against hyperlipidemia

Xiao-lei Yu[a, b], Xin Meng[b], Yi-Di Yan[b], Hui Wang[b], Lei Zhang*[a]

a MOE Key Laboratory for Nonequilibrium Synthesis and Modulation of Condensed Matter, School of Physics, Xi'an Jiaotong University, No. 28, Xianning West Road, Xi'an, Shaanxi Province 710049, People's Republic of China

b Meat Processing and Safety control professional technology innovation center, Jinzhou Medical University, No. 40, Section 3, Songpo Road, Langhe District, Jinzhou, Liaoning Province 121000, People's Republic of China

* Correspondence: Lei Zhang: zhangleio@mail.xjtu.edu.cn

MOE Key Laboratory for Nonequilibrium Synthesis and Modulation of Condensed Matter, School of Physics, Xi'an Jiaotong University, No. 28, Xianning West Road, Xi'an, Shaanxi Province 710049, People's Republic of China

Tel: +86-029-82668634

Tables captions

Table S1. Reaction system for fluorescence quantitative PCR.

Table S2. Gene primer sequences.

Table S1. Reaction system for fluorescence quantitative PCR.

Reagent	Volume (μl)
PerfectStart® Green qPCR SuperMix	10
Primer	1.5
cDNA	1
Nuclease-free Water	7.5
Total volume	20

Table S2. Gene primer sequences.

Primer	Forward (5′–3′)	Reverse (5′–3′)
FAS	AGTGTCCACCAACAAGCG	GATGCCGTCAGGTTTCAG
PPAR*α*	CGAAGACAAAGAGGCAGAGGT	AAGGAGGACAGCATCGTGAAG
SREBP-1c	GCGCCATGGACGAGCTG	TTGGCACCTGGGCTGCT
β-actin	GGTCATCACTATTGGCAACG	TCCATACCCAAGAAGGAAGG

Figures captions

Fig. S1. Ultraviolet absorption spectrum of naringin refined products and their standards.

Fig. S2. Fourier transform infrared spectrum of naringin.

Fig. S3. ^{1}HNMR spectrum of naringin products.

Fig. S4. ^{1}HNMR spectrum of naringin standard.

Fig. S5. ^{13}C NMR spectrum of naringin products.

Fig. S6. ^{13}C NMR spectrum of naringin standard.

Fig. S7. Mass spectrum of naringin products.

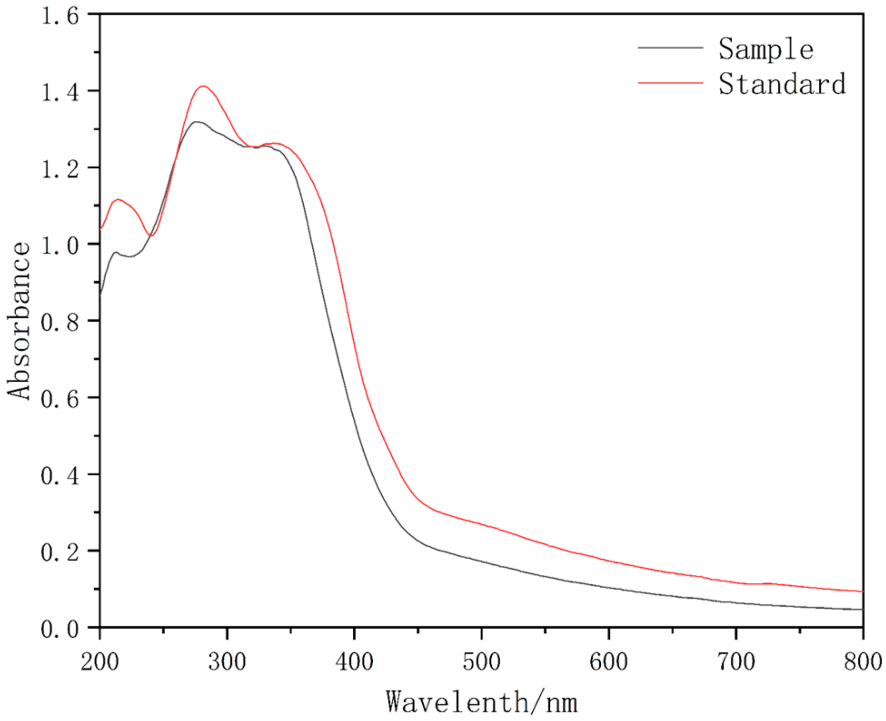

Figure S1. Ultraviolet absorption spectrum of naringin refined products and their standards（The UV absorption peak of naringin appears at 280 nm and 334 nm）.

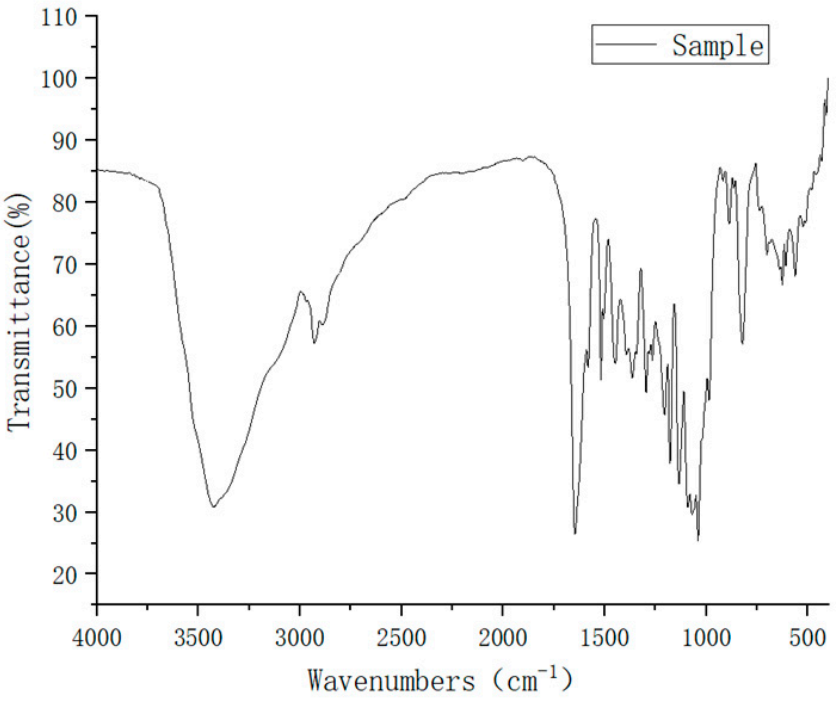

Figure S2. Fourier transform infrared spectrum of naringin（It shows the strong and wide absorption peak of 3180-3680 cm^{-1}）.

Figure S2 shows the strong and wide absorption peak of 3180-3680 cm^{-1} in the infrared spectrum that is attributed to the alcohol hydroxyl group and multiple phenolic hydroxyl groups in naringin. The absorption peak of 3000—3300 cm^{-1} is attributed to the C-H stretching vibration on the benzene ring. The absorption peak of 2850—2950 cm^{-1} is attributed to the C-H bond stretching vibration on the saturated carbon in the structure. Generally, the carbonyl stretching vibration absorption peak of flavonoids is approximately 1650 cm^{-1}, and the carbonyl stretching vibration absorption peak of dihydroflavonoids is approximately 1695 cm^{-1}. However, the hydroxyl group at position 5 in naringin forms an intramolecular hydrogen bond with the carbonyl group, which makes the absorption peak move to a lower frequency. The carbonyl stretching vibration at position 4 in the final naringin appears at 1647 cm^{-1}, 1581 cm^{-1}, 1518 cm^{-1}, and 1447 cm^{-1}, which are attributed to the C=C stretching vibration of the aromatic ring in the structure. The 1369 cm^{-1} and 1296 cm^{-1} peaks are attributed to the in-plane bending vibration of methylene in the structure. The multiple absorption peaks at 1208-1035 cm^{-1} are attributed to the C-O stretching vibration of aromatic ether and fatty ether bonds in the structure. The glycosidic bond absorption peak at 885 cm^{-1} indicates that the glycosidic bond is β-D-pyranoside, consistent with the structure. The absorption peak at 818 cm^{-1} is attributed to the C-H out of plane bending vibration of the B-ring aromatic ring para substituted structure.

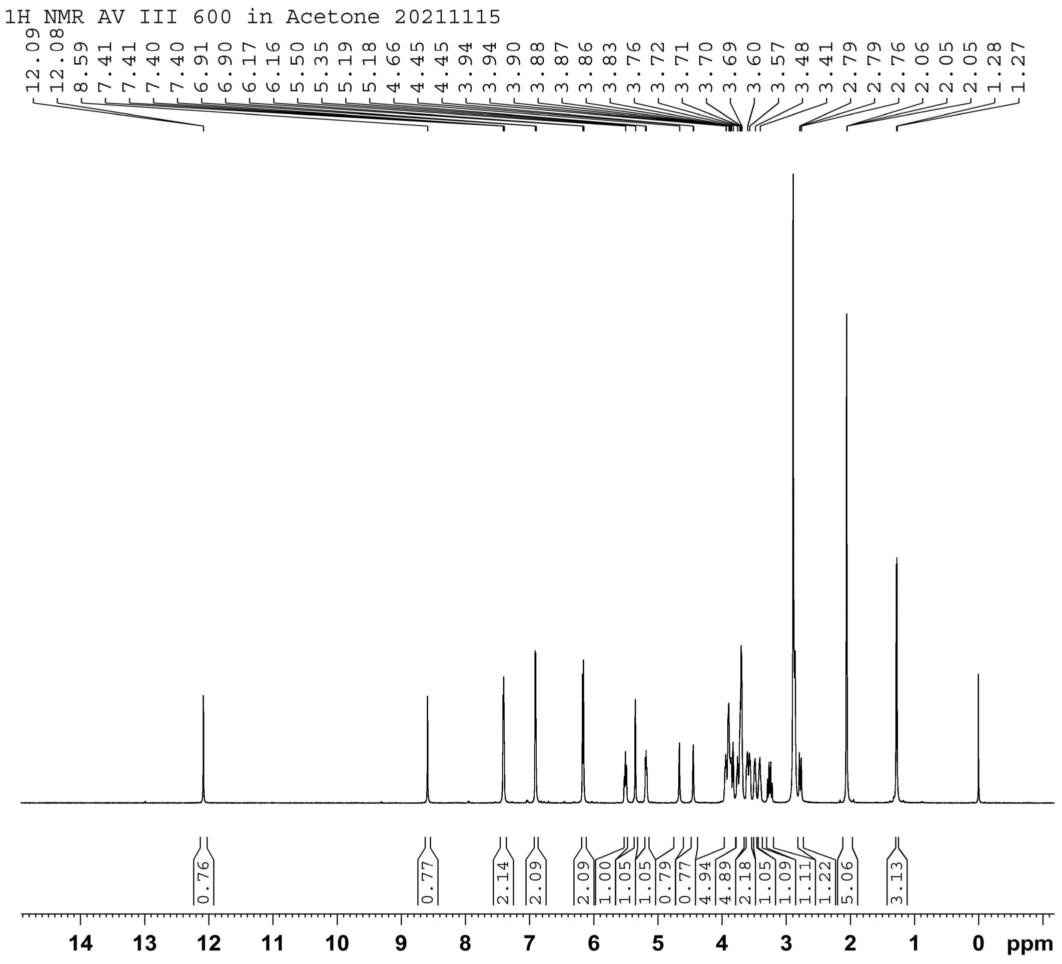

Figure S3. ^{1}HNMR spectrum of refined naringin products (400 MHz, Acetone-d_6).

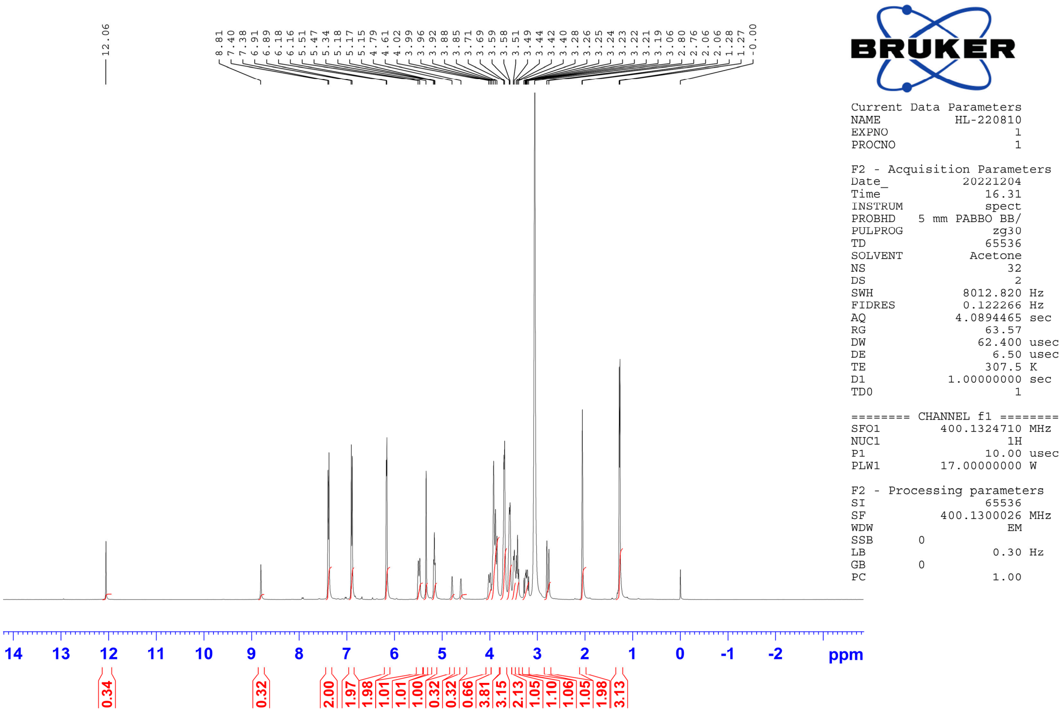

Figure S4. ^{1}H NMR spectrum of naringin standard (400 MHz, Acetone-d_6).

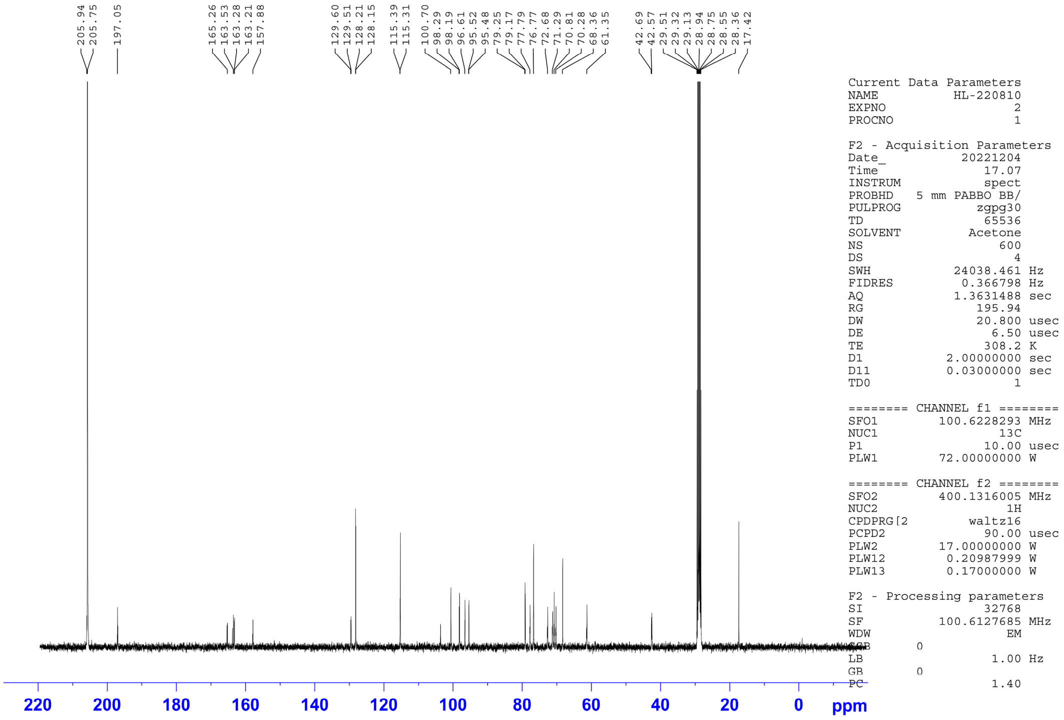

Figure S5. ^{13}C NMR spectrum of refined naringin products (400 MHz, Acetone-d_6).

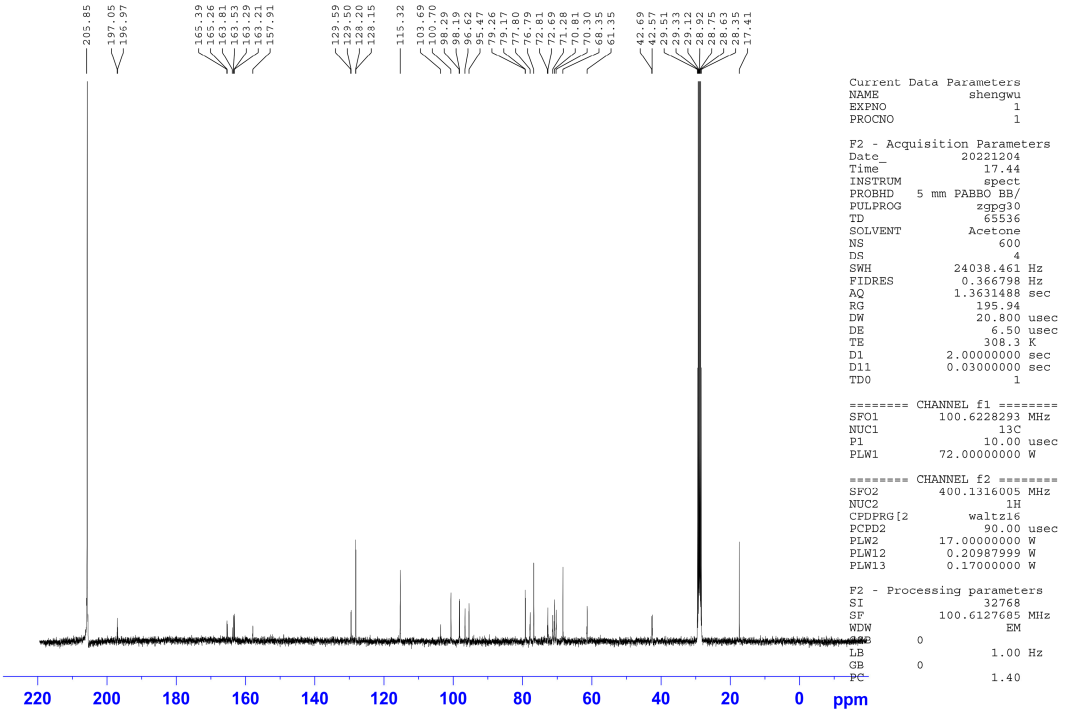

Figure S6. ^{13}C NMR spectrum of naringin standard(400 MHz, Acetone-d_6).

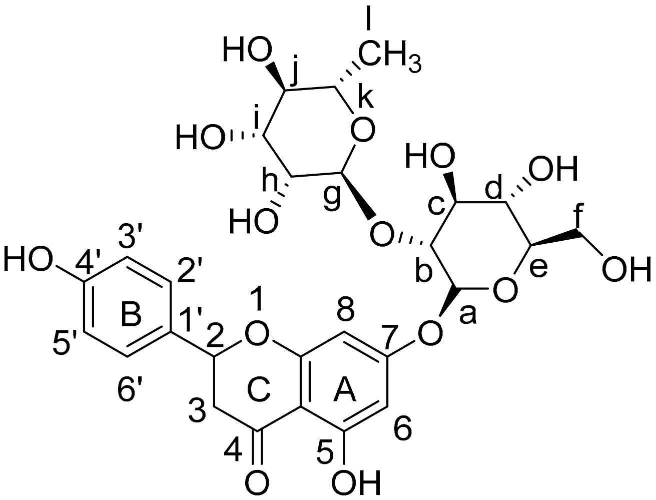

As shown in Figure S3-S6, the ^{1}H NMR and ^{13}C NMR of our refined naringin products is highly consistent with that of the standard.

^{1}H NMR (400 MHz, Acetone-d_6): δ 12.09 (s, 1H), 8.59 (s, 1H), 7.40-7.42 (dd, *J*=2.72, 8.3 Hz, 2H), 6.90-6.91 (d, 8.11, 2H), 6.16-6.17 (d, *J*=9.27, 2H), 5.48-5.52 (t, 1H), 5.35 (s, 1H), 5.17-5.19 (dd, *J*=7.09, 10.43 Hz, 1H),

4.66 (s, 1H), 4.44-4.45 (d, *J*=4.44, 1H), 3.83-3.95 (m, 4H), 3.69-3.77 (m, 4H), 3.56-3.61 (m, 2H), 3.48-3.49 (m, 1H), 3.41-3.42 (m, 1H), 3.22-3.29 (dd, 1H), 2.86 (s, 2H), 2.76-2.79 (dt, 1H), 1.27-1.28 (d, *J*=5.89, 3H). ^{13}C NMR (400 MHz, Acetone-*d6*): δ 205.75, 197.13, 197.05, 196.97, 165.39, 165.26, 163.81, 163.53, 163.29, 163.22, 157.88, 129.61, 129.51, 128.21, 128.15, 115.39, 115.31, 103.69, 100.70, 98.29, 98.19, 96.62, 95.52, 95.48, 79.26, 79.17, 77.79, 76.77, 72.81, 72.69, 71.29, 70.92,70.81, 70.29, 68.36, 61.47, 61.35,42.70,42.57,17.42.

Of this, δ = 12.09 and 8.59 belong to the hydrogen of phenolic hydroxyl group at positions 5 and 4, respectively. Since the oxygen atom and benzene ring form a conjugated large π bond, the hydrogen nucleus of phenolic hydroxyl group is affected by the electron absorption of oxygen atom and the conjugated π bond, the electron cloud density is further reduced, and the chemical shift appears at a higher level. Due to the effect of electron absorption of carbonyl group at position 4, the deshielding effect of the hydroxyl proton at position 5 is stronger than that at position 4', and the chemical shift is higher. δ = The DD peak of 7.40-7.42 belongs to the hydrogen on 2' and 6' of the B ring, δ = The D peak of 6.90-6.91 belongs to the hydrogen on 3' and 5' of the B ring. As the electron cloud density of the ortho atoms (3' and 5') of hydroxyl is higher than that of the meta atoms (2' and 6'), the hydrogen shielding effect on 3' and 5' is strong and appears at relatively low chemical shifts. δ = The D peak of 6.16-6.17 belongs to the hydrogen at positions 6 and 8 of the α ring. Due to the electron donor conjugation effect of the three oxygen atoms on the benzene ring, the electron cloud density at positions 6 and 8 is increased; hence, their shielding effect is stronger than that of other hydrogen nuclei on the benzene ring, and their chemical shifts are lower. δ = 5.5 and 5.35 belongs to the hydrogen at the end of “a” position and “g” position, respectively. The coupling constants at the “a” site are all about 8, indicating that the configuration of the terminal carbon atom here is

β-Glycoside bond, which is consistent with the conclusion of infrared spectrum and the structure of naringin; The type of glycosidic bond at the G position cannot be judged by the coupling constant, because the glycosyl structure here is rhamnose, and the hydrogen at the H position is always on the transverse bond. The peak at δ = 5.18 belongs to the hydrogen at position 2 on the C ring. Two hydrogen atoms belonging to the methylene group at "f" at 3.70-3.52. At d=3.94 and d=2.76-2.79, the two hydrogens belonging to the methylene group at the 3 position of the C ring have different chemical environments; hence, their chemical shifts are quite different, which is also consistent with the reports in literature (Study on the chemical composition and activity of Huyou peel). δ = From 1.27-1.28, three hydrogens on methyl group in rhamnose fraction. The peaks at other positions belong to the hydrogen on the methylene group of the sugar group and the hydrogen on the hydroxyl group of the alcohol, is not repeated.

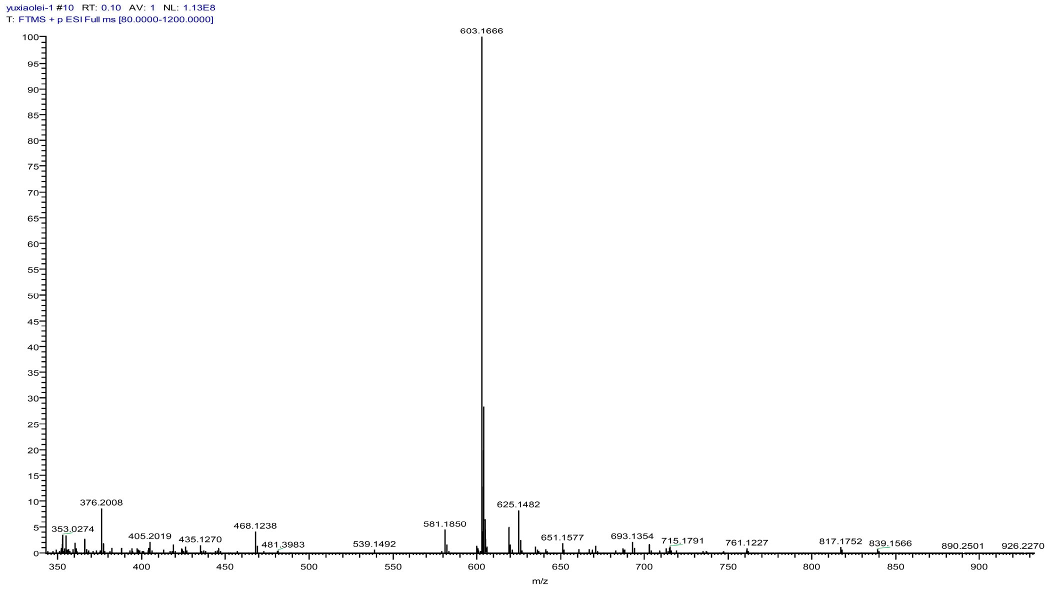

Figure S7. Mass spectrum of refined naringin products（electrospray ionization (ESI) can obtain the molecular ion peak of the compound）.

As shown in Figure S7, electrospray ionization (ESI) can obtain the molecular ion peak of the compound. As shown in the figure, *m/z*=581.1860 is the $[M + H]^+$ peak of naringin; *M/z*=603.1666 is the $[M + Na]^+$ peak of naringin; *m/z* =619.1412 is the $[M + K]^+$ peak of naringin. During ESI, some molecules can also break chemical bonds. *m/z* =435.1270 in the figure is the fragment peak $[M+H]^+$ of naringin-7-o-glucoside generated from the removal of rhamnose from naringin molecules during ionization, which further proves the correctness of the structure.
